# Chitosan-Based Polymer Nanocomposites for Environmental Remediation of Mercury Pollution

**DOI:** 10.3390/polym15030482

**Published:** 2023-01-17

**Authors:** Mvula Confidence Goci, Anny Leudjo Taka, Lynwill Martin, Michael John Klink

**Affiliations:** 1 Department of Biotechnology/Chemistry, Vaal University of Technology, Andries Potgieter Boulevard, Vanderbijlpark 1911, South Africa; 2 Cape Point Global Atmosphere Watch Station, South African Weather Service, c/o CSIR, Stellenbosch 7599, South Africa; 3 Chemical Resource Beneficiation, Atmospheric Chemistry Research Group, North-West University, Potchefstroom 2520, South Africa

**Keywords:** mercury pollution, chitosan, polymer nanocomposites, nanomaterials, passive sampler, batch adsorption

## Abstract

Mercury is a well-known heavy metal pollutant of global importance, typically found in effluents (lakes, oceans, and sewage) and released into the atmosphere. It is highly toxic to humans, animals and plants. Therefore, the current challenge is to develop efficient materials and techniques that can be used to remediate mercury pollution in water and the atmosphere, even in low concentrations. The paper aims to review the chitosan-based polymer nanocomposite materials that have been used for the environmental remediation of mercury pollution since they possess multifunctional properties, beneficial for the adsorption of various kinds of pollutants from wastewater and the atmosphere. In addition, these chitosan-based polymer nanocomposites are made of non-toxic materials that are environmentally friendly, highly porous, biocompatible, biodegradable, and recyclable; they have a high number of surface active sites, are earth-abundant, have minimal surface defects, and are metal-free. Advances in the modification of the chitosan, mainly with nanomaterials such as multi-walled carbon nanotube and nanoparticles (Ag, TiO_2_, S, and ZnO), and its use for mercury uptake by batch adsorption and passive sampler methods are discussed.

## 1. Introduction

Mercury (Hg) is one of the most toxic trace elements released into the atmosphere and is regarded as one of the “ten prominent chemicals of concern” due to its hazardous effects on human health and the environment [1,2]. Mercury is generally found in geological formations as sulfide ore (cinnabar–HgS), while as a trace element it is also found in other naturally occurring deposits, e.g., coal [3]. Special properties of mercury include its high vapor pressure; unlike other heavy metals, mercury can be vaporized into the surrounding air at low temperatures. It occurs in the atmosphere in three main forms; gaseous elemental mercury (GEM), gaseous oxidized mercury (GOM), and particulate-bound mercury (PBM), of which GEM is the predominant form in the atmosphere, accounting for 95–99% of all mercury in the atmosphere [4,5,6].

Furthermore, mercury is found in wastewater as methylmercury, which is produced from inorganic mercury through methylation (a microbial process controlled by certain bacteria and enhanced by chemical and environmental variables such as the presence of organic matter). Methylmercury affects many water bodies that do not have obvious sources of mercury, and this is because mercury emissions travel far into the atmosphere before being deposited on the Earth’s surface [6,7]. The toxicity of methylmercury is of concern because it is highly soluble in water. Even at low concentrations in drinking water, it can damage the central nervous system [8]. There is a huge and rapidly growing area of scientific literature on the distribution of mercury in multiple ecosystems. The atmosphere is the main transport route for Hg emissions, while soil and water play significant roles in mercury redistribution in multiple ecosystems [9]. Once mercury is released into the atmosphere, it can be transported in its elemental form [Hg (0)]. The predominant route for this elemental mercury is deposition in soil or water after oxidation to divalent mercury [Hg(II)] [10]. Mercury deposited on land surfaces is mainly taken up by soil and vegetation, but can also enter water bodies through drainage, runoff and erosion processes [11]. When inorganic mercury compounds are deposited in water and/or soil, they undergo microbial metabolism and are attenuated to methylmercury, which has the ability to bioaccumulate and concentrate in the food chain, particularly in fish and marine mammals. In addition, mercury has been shown to indirectly and directly affect human health and aquatic biota due to its toxicity and carcinogenicity. It also causes bad taste, color or odor problems in the water [12]. Anthropogenic events such as fuel use and artisanal gold mining discharge high concentrations of mercury into the air, soil, and water [13]. Like other heavy metals, mercury cannot be degraded in ecosystems, so remediation should be based on removal or control processes. Thus, the World Health Organization (WHO) and the United States Environmental Protection Agency (USEPA) have set the maximum permissible Hg concentrations in drinking water at 0.002 mg L^−1^ and 0.001 mg L^−1^, respectively [14,15].

There are numerous techniques including adsorption, solvent extraction, chemical precipitation, membrane filtration, solvent extraction, and ion exchange to remove mercury from wastewater and atmosphere. Table 1 presents a summary of these techniques with their advantages and limitations

The main objective of these techniques is to remove mercury from contaminated media (water or atmosphere) or to convert toxic types of mercury into less toxic ones in order to comply with the permissible limits required by law [24]. Regardless of the fact that these methods are effective, they are expensive, with high sludge production and by-product formation [25]. One of the most promising techniques is adsorption due to its affordability, high efficiency, and minimal use of chemicals, process flexibility, and ease of implementation in wastewater treatment plants. For these purposes, selective, economical, and ideal materials or adsorbents with high adsorptivity have to be developed [25].

Almost all previous studies found that the main disadvantage of the uptake rate of mercury from wastewater is the limited surface area of the adsorbent and complicated conjugation chemistries. A passive mercury sampler was affected by meteorological factors such as temperature, humidity and wind speed [26,27,28]. In addition, these conventional adsorbents are not environmentally friendly and have demonstrated poor recyclability, require a significant number of sample preparation steps and large volumes, limiting their application as adsorbents and passive samplers for mercury removal. Furthermore, these adsorbents have shown limitations such as their high operational cost and incapability to remove mercury contaminants from wastewater and atmosphere to acceptable concentration levels (0.002 mg L^−1^ and 0.001 mg L^−1^). The search for an ideal adsorbent material, which is cheap, inexpensive, earth-abundant, green, and is capable of selectively trapping mercury, remains a challenge. 

Hence, nanotechnology is currently regarded as the most promising method for water decontamination and removal of environmental pollutants. The application of nanotechnology in environmental decontamination involves the use of nanomaterials as adsorbent materials which are called nano sorbents to remove or adsorb the pollutants from water or air. These nano sorbents are nanostructured materials with pore sizes between 1 and 100 nm onto which the pollutant molecules (inorganics, organics, antimicrobials, pathogens, and microorganisms can be adsorbed [29,30]. Among the various nanomaterial adsorbents, chitosan-based polymer nanocomposite materials have attracted great research attention because they possess multifunctional properties, useful for the adsorption of various kinds of pollutants from wastewater and the atmosphere. In addition, these chitosan-based polymer nanocomposites are made of non-toxic or less toxic materials that are environmentally friendly, highly porous, biocompatible, biodegradable, and recyclable. They have a high number of surface active sites, are earth-abundant, have minimal surface defects, and are metal-free. These green properties of chitosan-based polymer nanocomposites make them efficient nano sorbent materials for the environment [31,32,33,34]. Such biopolymer-based nanocomposites have emerged as a promising adsorption material for the uptake of mercury from wastewater and the atmosphere. These biopolymer-based nanocomposite materials (e.g., chitosan-based polymer nanocomposites) have attracted much research attention. They consist of several phases, with one of the phases containing additives in the nano range. They also possess excellent multifunctional properties resulting from the combination of the individual components. These biopolymer-based nanocomposites have been shown to improve adsorption efficiency due to the electron-rich functional groups present on the polymer backbone [35].

Therefore, the focus of this paper is to review the advances in the modifications of chitosan mainly with nanomaterials such as multi-walled carbon nanotube and nanoparticles (Ag, TiO_2_, S, and ZnO) to obtain chitosan-based polymer nanocomposites. Additionally, the potential of these chitosan-based polymer nanocomposites, respectively, as adsorbents in batch adsorption techniques for the removal of mercury from contaminated water and as passive samplers for gaseous mercury removal from the atmosphere, is discussed.

## 2. Mercury as an Environmental Pollutant and Occurrence in the Atmosphere

Mercury is identified by the symbol Hg, derived from hydrargyrum, its Latin name, meaning silver water [36]. In its elemental form, it exists as a silver liquid at room temperature, since Hg has a melting point of −38.87 ℃ [36,37]. Mercury occurs naturally in the environment, with more than 25 Hg-containing minerals known to occur in the Earth’s mantle [37]. The most abundant Hg-bearing ore is cinnabar (HgS) [36,38,39]. Mercury as an environmental pollutant poses a global threat due to its known toxicology and its ability to bioaccumulate in ecosystems [4,40]. Humans are susceptible to this potent toxin, mainly through eating contaminated fish, which can cause severe neurological defects [41,42]. In addition, mercury is very volatile and exists in the gaseous state in the atmosphere and is easily reduced to the elemental form (Hg), thus being relatively unreactive and not very soluble in water. These particular properties allow mercury to have an atmospheric residence time of around 6–12 months, which could allow mercury to travel thousands of kilometers before being removed through either wet or dry deposition, thus acting as a global pollutant [4,6]. 

Additionally, mercury is a naturally occurring metal that combines with other elements to form inorganic pollutants found in wastewater. Mercury deposited on land surfaces is mainly taken up by soil and vegetation, but can also enter water bodies through drainage, runoff and erosion processes [11]. When inorganic mercury compounds are deposited in water and/or soil, they undergo microbial metabolism and are attenuated to methylmercury, which has the ability to bioaccumulate and concentrate in the food chain, particularly in fish and marine mammals. In addition, mercury has been shown to indirectly and directly affect human health and aquatic biota due to its toxicity and carcinogenicity. It also causes bad taste, color or odor problems in the water [12].

The global total of mercury pollutant released into the atmosphere is estimated at about 7527 t.y-1 [43]. South Africa is currently considered the sixth largest emitter of mercury worldwide [43], with estimated anthropogenic emissions between 27.9 t.y-1 and 50 t.y-1 [44,45,46,47]. In 2013, South Africa became a signatory to the Minamata Convention on Mercury, a global agreement to reduce global mercury emissions [47]. Therefore, it is expected that mercury could be considered a critical pollutant in South Africa’s National Ambient Air Quality Standards (NAAQS) in the near future [48]. According to the United Nations Environment Program (UNEP) report (UNEP, 2018), global mercury emissions from anthropogenic sources to air amounted to about 2220 tons in 2015. Among anthropogenic sources, stationary burning of fossil fuels accounts for 24% of the estimated emissions, mainly from coal burning (21%). It is estimated that the concentration of mercury in the atmosphere has increased 3 to 5 times over the past century as a result of anthropogenic activities and has tripled in the surface waters of the oceans [49]. Streets et al. (2019) estimated that global anthropogenic mercury emissions increased from about 2188 t in 2010 to about 2390 t in 2015 (9.2% increase) [50]. 

The literature discloses several types of adsorbents that have been studied for mercury concentration monitoring or mercury remediation in wastewater or in the atmosphere. Conventional adsorbents such as zeolites [51], clay minerals [52], natural and modified bentonite [53], and impregnated activated carbon [28], have been used to remove mercury pollution in wastewater and gaseous mercury in the atmosphere. For example, researchers from Peking University and the University of Toronto used sulfur-impregnated activated carbon for a passive mercury sampler [28,54]. These materials, although effective adsorbents, are cost-prohibitive. Abbas et al. (2018) synthesized a novel mesoporous conjugated adsorbent based on pentasil zeolite (ZSM-5 type) for adsorption of mercury in aqueous solution, and the maximum adsorption capacity reached 172.6 mg/g. Bao et al. (2017) used silica-coated magnetic nanoparticles to extract mercury from wastewater. Li and co-workers have designed and implemented ZnS nanoparticles (NPs) in various approaches to remove mercury from polluted environments [55].

## 3. Chitosan and its Modifications

**Figure 1:**
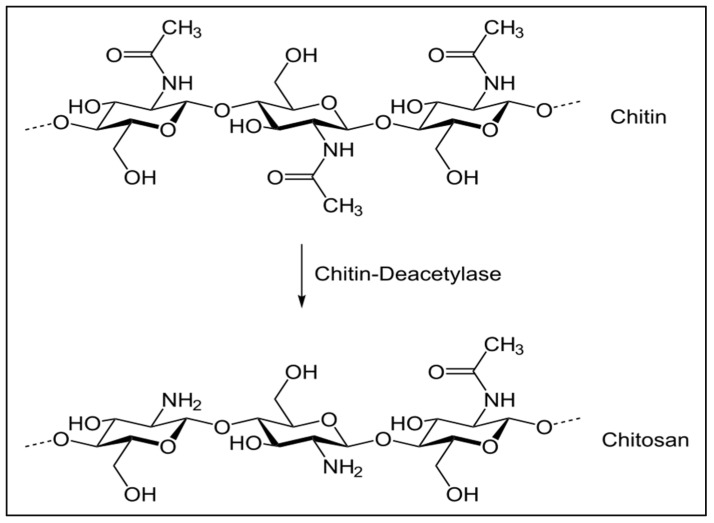
Formation of chitosan by partial deacetylation of chitin.

Chitosan is a partially deacetylated polymer derived from the fundamental deacetylation of chitin, an unbranched, glucose-based polysaccharide that is abundant in the major components of cell-wall of fungi, crustaceans, and insect exoskeletons, as well as some bacterial and fungal cell walls [56,57,58]. Chitosan consists of linear β-(1, 4)-linked N-acetyl-glucosamine units as shown in Figure 1. The quality of chitosan depends on the source of chitin and its separation and degree of deacetylation [59].

In addition, depending on the origin of the polymer and treatment during the extraction process, chitosan shows crystallinity and polymorphism. Chitin, or 0% deacetylated and fully deacetylated chitosan, or 100% deacetylated, had the highest crystallinity. In acidic environments, chitosan with straight, unbranched morphology and increased molecular content enhances viscosity [60]. The chemical structure of chitosan compared to other polysaccharides (cellulose or starch) allows particular modifications due to a large electrostatic attraction mechanism to build polymers for specific applications [61]. Chitosan polymers are non-toxic, antimicrobial, biodegradable, biocompatible, and they are natural amino-polysaccharides with unique structures, multidimensional properties, excellent functions and wide-ranging applications in biomedical and other industrial fields [34].

However, in most organic solvents and water at neutral pH, chitosan is insoluble in its original form (pristine). This insolubility of pristine chitosan limits its applications to some extent [58,62]. Hence, physical or chemical modification is crucial to improve solubility (over a wide pH range in water and organic solvents) and develop properties useful for further chemical reactions and applications [63]. Therefore, various modification methods have been developed such as physical and chemical modifications [64,65]. Functionalization (or modification) of chitosan has been demonstrated to introduce the desired mechanical, chemical, and physical properties, which are vital for enhancing its reactivity with nanomaterials in further reactions [56,58,64,65,66,67,68,69].

Chemical methods involve the direct preparation of the inorganic nanoparticles (NPs) (metal oxide and metal) in the polymer matrix solution used as the reaction medium. Chemical methods involve techniques such as cross-linking, surface grafting, and introducing coordination atoms with different supporting materials [56,58,65,67,70]. For instance, uniform size and shape of chitosan-based polymer nanocomposites are often obtained when using chemical methods. This chemical modification is beneficial for their use in water purification and environmental remediation. Physical methods first necessitate the preparation of the NPs, followed by their addition to the polymer matrix used as the dispersion medium [63,71]. For example, functionalization of chitosan using physical methods includes ultraviolet irradiation, electron beam irradiation, ultrasonication, physical mixing, Y-ray irradiation, mixing, plasma irradiation, sputtering, and coating processes [67]. In addition, this method includes the fabrication of beads, membranes, and chitosan nanocomposite films [63]. Among these two techniques, chemical methods are preferred because during the preparation of the polymer nanocomposites, the polymer matrix helps to control the size and shape of the NPs by acting as a capping agent or stabilizer to avoid agglomeration of NPs [31,72]. Moreover, the chemical modification of chitosan to produce new functionalized chitosan-based polymer composite materials is of primary interest because such procedure would generate novel properties and functions of functionalized chitosan favorable for their use in various applications especially in water treatment and removal in gaseous mercury from the atmosphere [33]. 

Depending on the nature of the polymer, various techniques can be used to effect crosslinking. Crosslinking can occur through polymerization of monomers with more than two functionalities (through condensation) or through covalent bonding between polymer chains through irradiation or chemical reactions by the addition of various chemicals in conjunction with heating and sometimes pressure [73]. The addition of crosslinking between polymer chains affects the physical properties of the polymer depending on the degree of crosslinking and the presence and absence of crystallinity. Crosslinking leads to elasticity, decrease in viscosity, insolubility of the polymer, increase in strength and toughness, decrease in melting point and conversion of thermoplastics to thermosets [31].

## 4. Progress on the Synthesis of Chitosan Functionalized with Nanomaterials

The research progress in the synthesis of chitosan-based polymer nanocomposites has attracted a high level of awareness due to their intriguing chemical and physical properties that offer various potential applications. In particular, chitosan-based polymer nanocomposites containing carbon nanotubes and/or NPs such as Ag, TiO_2_, S, and ZnO fixed in the biopolymer matrix have been developed for their use as adsorbents to remove mercury from wastewater and the atmosphere [74,75,76].

These chitosan-based polymer nanocomposites have shown to improve adsorption and degradation of pollutants, as well as having antibacterial activity. This can be proved via the excellent mechanical and physico-chemical properties offered by these nanoparticles and carbon nanotubes, which are also valuable for enhancing the properties of chitosan [27,33,77,78,79,80,81]. For instance, functionalized chitosan modified with nanomaterials to obtain chitosan-based nanocomposites improve chemical stability, diffusion properties, surface area, number of adsorption sites and porosity, as well as adsorption capacity and reusability [31,34,80,81,82,83]. Table 2 presents a properties comparison of different adsorbents with their advantages and disadvantages.

### 4.1. Chitosan Modified with Carbon Nanotubes

**Figure 2:**
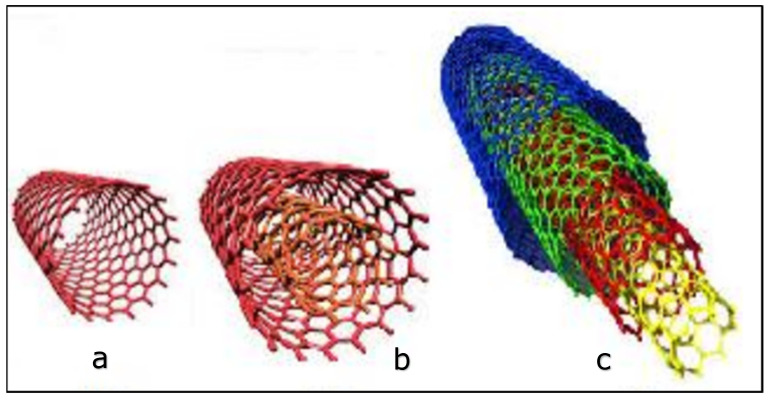
Types of carbon nanotubes: single-walled carbon nanotube (SWCNTs (**a**)), double-walled carbon nanotube (DWCNTs (**b**)) and multiple-walled carbon nanotube (MWCNTs (**c**)) [94].

Carbon nanotubes (CNTs) were discovered by Lijima in 1991 [89]. Carbon nanotubes are carbon allotropes (which include diamond, graphite, and graphene) and emerged in the field of nanotechnology because of their nano-size and unique properties. They are of great interest due to their simplicity and easy synthesis [90]. In general, CNTs are classified into three main types: single-walled carbon nanotubes (SWCNTs), double-walled carbon nanotubes, and multi-walled carbon nanotubes (MWCNTs). SWCNT is defined as a layer of graphene sheet rolled into a single cylinder, DWCNT is a two-layer graphene sheet rolled into a double cylinder, while MWCNT is a multiple-rolled layer of graphene sheets as shown in Figure 2. These rolled graphene sheets are held together by van der Waals interactions, which cause CNTs to bundle together and lead to the formation of large aggregates [91,92,93].

**Figure 3:**
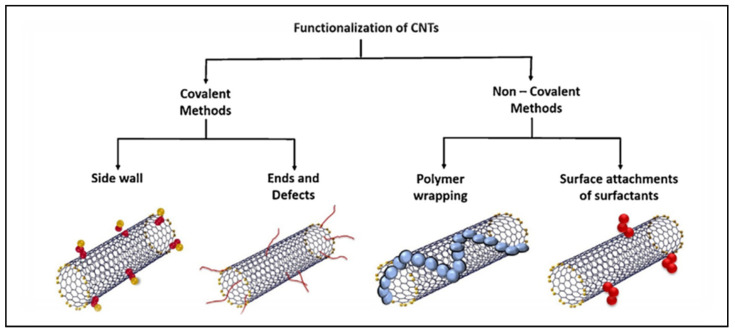
Functionalization methods of carbon nanotubes [99], with permission from Elsevier, 2022.

CNTs can be produced by laser ablation, arc discharge, and chemical vapor deposition (CVD) [95,96,97]. The CNTs, when produced, are insoluble and less dispersive substances. Therefore, it is essential to improve their surface properties for enhanced solubility in most solvents, increased chemical reactivity, biocompatibility, and reduced cytotoxicity [33,34,96]. Functionalization of CNTs can be achieved using various approaches such as covalent and non-covalent methods, as illustrated in Figure 3. For example, the functionalization by covalent method can be conducted by acid treatment which favours the introduction of functional groups such as carbonyl, hydroxyl, and carboxylic groups on the surface of CNTs. These functional groups are useful for the further modification of CNTs with other chemical moieties or materials (eg. chitosan) [31,93,96,98].

Therefore, to overcome the limitations of CNTs, a surface modification process is essential to improve their surface properties by changing the surface of the materials [100]. For instance, the properties of functionalized CNTs make them good adsorbents for the selective removal of specific pollutants.

Modified CNTs have gained recognition as attractive adsorbents for wastewater treatment and environmental remediation applications. This is due to their remarkably high sorption capacity resulting from the efficiency, affinity and interaction between CNT surface’s functional groups and pollutants [101,102]. In addition, CNTs have high surface-to-volume ratio, uniform pore distribution, and highly porous and hollow structures which make them good candidates as superior adsorbents for removal of heavy metal ions [103,104] dyes [105], and organic pollutants [102] from aqueous solution.

Carbon nanotubes, especially multi-walled carbon nanotubes (MWCNTs), are known as superior adsorbents and have been used as one of the components in chitosan-based polymer nanocomposites. Because of their excellent mechanical, electronic, and optical properties, MWCNTs are among the most widely studied and synthesized new materials [106]. Chitosan-CNTs based nanocomposites have attracted great interest in a variety of research activities due to their high adsorption capacities [106,107,108,109,110,111]. For example, the strength imparted to the polymer nanocomposite by MWCNT ensures good stability, supports good adsorption capacities at low concentration, and improves the recyclability and recovery of the synthesized polymer [112].

Salam and co-workers synthesized homogeneous MWCNTs/chitosan nanocomposites by cross-link polymerization of MWCNTs using glutaraldehyde as a cross-linking agent. The synthesized MWCNTs/chitosan nanocomposites were evaluated for the uptake of metal ion impurities (Cd, Cu, Zn, Hg, and Ni) from aqueous solution by column adsorption. Their study proved that MWCNTs/chitosan nanocomposite can efficiently remove metal ions from aqueous solutions due to the beneficial effect of cross-linking MWCNTs with chitosan [78]. Mbianda and co-workers modified the oxidized CNTs obtained after acid treatment with an aminophosphonate together with amino-alcohol to produce phosphorylated CNTs (pCNTs) [107]. Zhu et al. (2013) report the fabrication of chitosan-modified magnetic graphitized multi-walled carbon nanotubes (CS-m-GMCNTs) using a suspension cross-linking method [113].

### 4.2. Chitosan Modified with Metal Nanoparticles: Silver, Titanium Dioxide, Sulfur, and Zinc Oxide

Nanoparticles have two key properties that make them particularly attractive as sorbents. On a mass basis, they have much larger surface areas than bulk particles. Nanoparticles can be classified as either organic or inorganic, and they can also be functionalized with different chemical groups to increase their affinity for target compounds [114]. Furthermore, depending on the functionalization or charges on the nanoparticle (NP) shells, ordered thin-film or 3D structures can also be designed by drop-casting, which is one of the simplest and cheapest deposition techniques [115], although it is rarely able to build up homogeneous layers, especially on large surfaces, mainly due to different evaporation rates through the substrate or fluctuations in concentration, which can lead to variations in the internal structure and film thickness. 

These nanoparticles have been used to remove heavy metal ions (chromium, mercury and lead), organic and micro-organism pollutants from wastewater [116,117]. However, the literature has demonstrated that using these NPs on their own as adsorbent materials usually results to agglomeration and is not environmentally green [118]. Hence, the immobilization of these NPs onto a carbon nanomaterial or polymer matrix (chitosan), before they can be used for environmental remediation, is crucial.

#### 4.2.1. Chitosan Modified with Silver Nanoparticles

**Figure 4:**
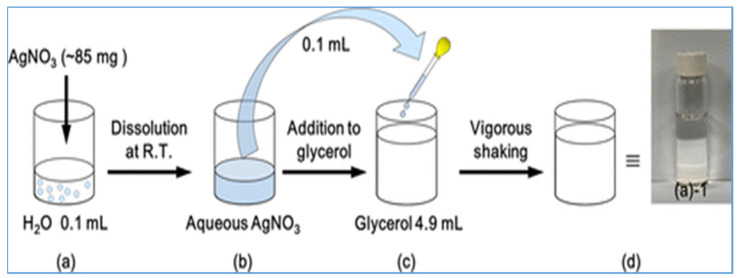
Chemical synthesis of silver nanoparticles with silver nitrate and glycerol under natural conditions [121].

Silver nanoparticles (AgNPs) have gained popularity due to their high stability and enhanced antimicrobial activities. This could be attributed to their small size and large surface-to-volume ratio, making them an improvement over their large counterparts. They have been shown to have effective antimicrobial activity even against resistant microbial strains at very low concentrations [119]. Hence, a variety of methods such as chemical, physical, photochemical, and biological methods have been employed to synthesize silver nanoparticles, which are recrystallized and purified [120]. For example, the chemical method is the most common method used to synthesize silver nanoparticles (as depicted in Figure 4), and it uses silver salt, stabilizer, and capping agent as the three main components to control the growth of Ag-NPs. Among these, silver nitrate is a silver salt that is widely used due to its chemical stability and low cost. 

The stabilizers include surfactants and ligands or polymers containing functional groups such as polyvinyl pyrrolidone, poly (ethylene glycol) and poly (methacrylic acid). In addition, polymers (e.g., collagen, chitosan) can also serve as stabilizers and capping agents for the synthesis of the polymer nanocomposites based silver nanoparticles [122]. For example, Al-Sherbini (2019) have prepared chitosan/poly (vinylidene chloride)/Ag (CS/PVDC/Ag) nanocomposite film using a chemical reduction method. The prepared CS/PVDC/Ag nanocomposite was used as an antibacterial agent against G. Bacillus and E. coli in water treatment. The synthesized chitosan/PVDC/Ag was also applied as a adsorbent for the removal of metal ions (Pb, Hg, Fe). Moreover, silver intercalation into the chitosan structure can boost antimicrobial efficacy and antibacterial action, opposing all types of bacteria [123]. Tyliszczak et al. (2017) synthesized hydrogel materials based on chitosan and modified with silver nanoparticles for measuring the swelling capacity and in vitro tests in distilled water. The study by Hani and Hui reported the synthesis of AgFeO_2_ and AgFeO_2_-modified chitosan (AgFeO_2_@CTS-NPs) using a hydrothermal method and was applied to the separation of biothiols [124]. 

#### 4.2.2. Chitosan Modified with Titanium Dioxide

**Figure 5:**
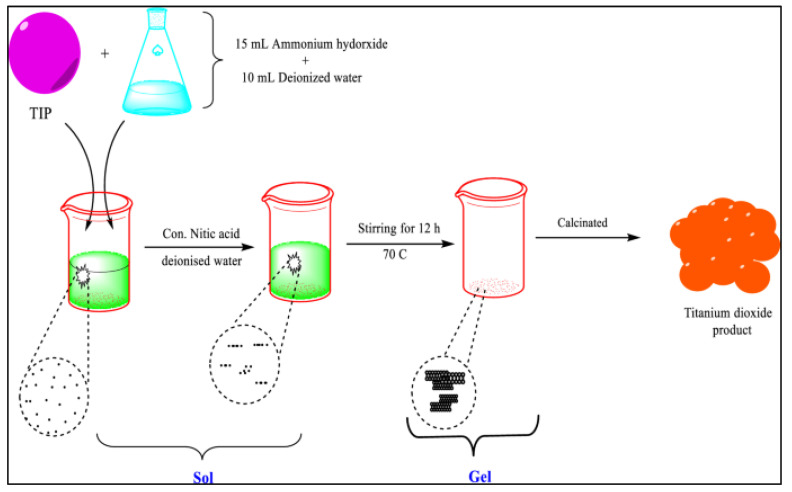
Preparation of TiO_2_ nanoparticles by sol-gel method [131], with permission from Springer Nature, 2022.

Titanium dioxide, also known as titania, is widely used as a photocatalyst for environmental remediation because it is environmentally friendly and has excellent photocatalytic and antimicrobial properties [125,126,127]. In addition, TiO_2_ mostly exists in three phases, namely anatase, rutile and brookite, with anatase being the most favourable dominant phase [126]. Among the nano photocatalysts used in the treatment of wastewater, TiO_2_ has been extensively studied; previous studies have shown that TiO_2_ nanoparticles have the potential to degrade wastewater contaminants [128]. Nowadays, the metal and metal oxide nanoparticles are synthesized by both chemical and physical methods such as hydrothermal [81], microwave [129], and sol-gel method [130]. Among all these synthesis techniques, the sol-gel method is mostly used for the synthesis of titanium dioxide as shown in Figure 5.

Moreover, new developments on the synthesis of chitosan-based TiO_2_ nanocomposites have been achieved by Bahal and co-workers. They have accomplished the synthesis of the chitosan-TiO_2_ nanocomposites using both chemical and physical methods. This synthesis was achieved as a result of a free radical polymerization reaction in the presence of potassium persulfate through grafting of acrylic acid unto chitosan. Then, the grafted acrylic acid/chitosan was modified with TiO_2_ nanoparticles by the ultrasonication technique [132]. 

Accordingly, Karthikeyan et al. (2017) reported the photocatalytic and antimicrobial activities of chitosan-TiO_2_ nanocomposite. It was effective against both Gram positive and Gram negative bacteria and a zone of inhibition between 10.333 ± 0.5773 and 25.667 ± 1.5275 (mm) was observed [133]. In a study by Dhanya and Aparna, they synthesized TiO_2_/chitosan-based hydrogel and almost completely removed azo and anthraquinone dyes from the wastewater [134].

#### 4.2.3. Chitosan Modified with Sulfur Nanoparticles

Sulfur nanoparticles (SNPs) constitute a chemically and biologically active element, with behaviours ranging from antioxidant action to antimicrobial properties [135]. In addition, sulfur and its abundance of chemically diverse organic and inorganic compounds are known to exhibit a broad and often diverse spectrum of biological activities, ranging from antioxidant effects to antimicrobial and even anticancer properties. SNPs are widely used as antimicrobial agents and are used in lithium-sulfur batteries and in sulfur-based photocatalysts, sulphuric acid production, and carbon nanotube modification [136,137,138]. 

**Figure 6:**
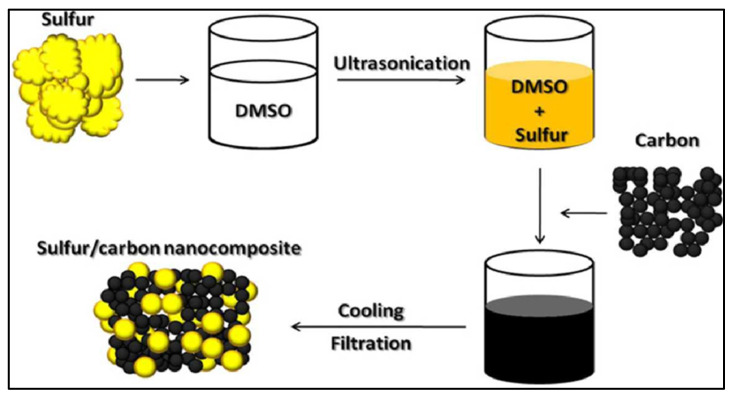
The synthesis process of sulfur nanoparticles [140].

Sulfur nanoparticles can be prepared by different methods such as acid hydrolysis of sodium thiosulphate, ultrasonic treatment of sulfur-cystine solution, and aqueous surfactant solutions [139,140]. Despite the existence of many exciting methods, there are only a few mentions in the recent literature that deal with the synthesis of sulfur nanoparticles. Deshpande et al. (2008) prepared sulfur nanoparticles from H_2_S gas using biodegradable iron chelate catalyst in reverse micro-emulsion technique, and Shankar et al. (2018) synthesized sulfur nanoparticles (SNPs) using chitosan as a capping and stabilizing agent [141,142]. Figure 6 illustrates the process of synthesizing sulfur nanoparticles, whereby dimethyl sulfoxide (DMSO) was used as the solvent to dissolve the sulphur.

Moreover, Xiurong Zhai et al. (2017) synthesized nitrogen, phosphorus, and sulfur co-doped porous carbons (N,P,S@PC) dispersed in the chitosan (CS) acid solution, particularly through covalent interaction, which could avoid N,P,S@PC aggregation in most solvents [143]. Shankal prepared sulfur nanoparticles (SNPs) using sodium thiosulfate and hydrochloric acid capped with chitosan to show potent antibacterial activity against *Escherichia coli* and *Staphylococcus aureus* [144]. Yuezhong Wen et al. (2015) developed carbonaceous sulfur-containing chitosan–Fe (III) for the removal of copper (II) from water. The adsorbent showed excellent copper removal performance due to its fast kinetic behaviour, high adsorption capacity and relatively good ability to withstand acidic conditions [145]. 

#### 4.2.4. Chitosan Modified with Zinc Oxide

Zinc oxide (ZnO) is mostly exploited in nano dimensions due to its exceptional scientific properties attributed to its band gap and large excitonic binding energy [146]. In addition, ZnO possesses very characteristic chemical and physical properties such as higher physicochemical stability, high chemical coupling ability, broad spectrum of radiation absorptivity and higher photostability [147,148]. It is commonly added to sunscreens, coatings and paints to absorb UV light and plays an important role in various industries. ZnO is a white to off-white crystalline powder that is nearly soluble in water. Its most common structures are wurtzite (hexagonal) and zinc blender [149]. 

Zinc oxide nanoparticles (ZnO NPs) can be derived or synthesized from various zinc salts using various techniques including vapour deposition, water precipitation methods, hydrothermal synthesis, sol-gel methods, and mechanical size reduction [150]. Among these techniques, sol-gel is mostly used due to many advantages such as low operational cost, and ease of the process, with reliability, and reproducibility of a similar product. The reaction also requires milder conditions for nanoparticle fabrication [151,152].

**Figure 7:**
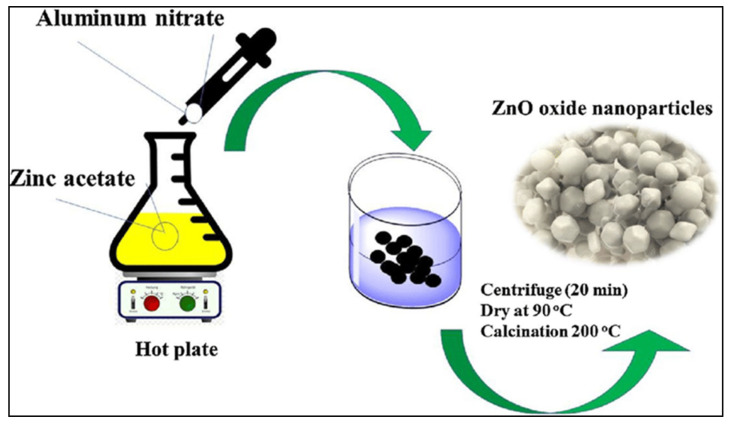
Preparation of zinc oxide (ZnO) nanoparticles by sol-gel method employing zinc acetate and aluminium nitrate as precursors [155].

Qiu and co-workers prepared nitrogen-doped ZnO for photocatalytic degradation of bisphenol under visible light [153]. Yue et al. (2013) synthesized ZnO NPs using the sol–gel technique with zinc 2-ethylhexanoate as precursor salt and Propan-2-ol as a solvent [148]. In addition, Taghavi Fardood et al. (2019) fabricated ZnO nanoparticles using zinc acetate and aluminium nitrate as precursors by a sol-gel method [154]. The aluminium nitrate was dissolved in zinc acetate drop wise, followed by centrifugation, drying, and calcination at 200 ℃, yielding zinc oxide nanoparticles as shown in Figure 7. 

Moreover, previous work has also reported on the modification of ZnO with chitosan. For instance, Hassan et al. reported the use of a chitosan-silica composite to immobilize zinc oxide (ZnO) nanoparticles into a chitosan/silica/ZnO nanocomposite [156]. Shahram Moradi Dehaghi and co-workers synthesized chitosan–ZnO nanoparticles (CS–ZnONPs) composite beads by a polymer-based method [157]. The study by Selvaraj Preethi et al. (2020) report an eco-friendly synthesis of chitosan/zinc oxide (CS/ZnO) nanocomposite using *S. lycopersicum* leaf extract by a bio-inspired method [158]. 

## 5. State of Art on Chitosan Modified with Nanomaterials for Environmental Remediation to Mercury Pollution

Table 3 summarizes recent work on the use of chitosan-based polymer for environmental remediation mainly focused on mercury. Based on the literature search, one can notice that since the past ten years, research studies on the synthesis of chitosan-based polymer nanocomposites, mainly resulting from the modification of chitosan with either CNTs, TiO_2_, Ag, ZnO or S nanoparticles, for application on environmental remediation to mercury have been scarce. Hence more work is still needed in this area.

The mechanism of adsorption of these heavy metal (Hg, Pb, Cu, and Cr) and inorganic contaminants turned out to be complex and the most commonly reported are: electrostatic attraction, physical adsorption (mainly due to van der Waal forces), adsorption-precipitation and chemical interaction (occurs between functionalized MWCNTs and metal ion pollutants). Additionally, the adsorption mechanism can also be studied using the kinetic models (first and second order pseudo-models) and the thermodynamic parameters such as the changes in enthalpy (H), entropy and Gibbs energy. It has also been reported that a combination of adsorption precipitation and electrostatic attraction can occur during the uptake of metal ions [111]. The study by Amanulla and co-worker, observed that besides the electron-hole pairs generated by light photons, hydroxide radicals also play an important role in the mercury degradation mechanism. The study indicates that the photocatalysts produced were very stable at room temperature and can be recycled and reused for up to four successful cycles without major loss of performance [160]. Moreover, the mechanism of adsorption of pollutants (mercury) by the adsorbents has also been studied using different adsorption isotherm models such as Langmuir, Freudlich, Redlich–Peterson, Dubinin–Radushkevich and Temkin models. This is because these isotherm models are essential for the design and operation of the adsorption system [111]. For instance, a study by Mahmoud et al. (2018) has successfully explained the sorption of divalent mercury and copper by three isotherm models (Langmuir, Freundlich and Dubinin–Radushkevich) and shown favorable operations. The nanocomposite retained its selectivity and sorption power for mercury and copper even in the presence of other interfering ions [164].

## 6. Adsorption Techniques for the Removal of Mercury from Contaminated Water and Atmosphere

Adsorption is defined as the transport of certain compounds (adsorbates or pollutants) from one phase (e.g., liquid) to adhere to the surface of another substance (e.g., solid adsorbent). It is a surface chemical process that occurs at the interface between adsorbent and adsorbate and mainly depends on the type of adsorbate, adsorbent and operating conditions. It is important to note that liquid-solid, gas-solid, gas-liquid, and liquid-liquid are the different interfaces normally involved in adsorption processes. However, in the case of water purification, only the liquid-solid interface is considered [165,166]. 

Currently, adsorption techniques have emerged as promising methods for water treatment and environmental monitoring due to their simplicity, lower cost, and potential for recycling the adsorbent materials [166,167]. Moreover, the adsorption process is considered to be the most feasible method because it is likely used to remove all types of pollutants including organic and inorganic matter from industrial wastewater, domestic wastewater, synthetic water, sewage, groundwater, and surface water including drinking water, because this method is competent, inexpensive, flexible in design, user-friendly and utilises a globally available adsorbent, with simple regeneration process of some used adsorbent and low energy consumption [168]. 

There are different types of adsorption techniques such as fixed-bed adsorption, batch adsorption, and passive samplers; and each adsorption technique has its advantages and disadvantages. For example, fixed bed adsorption is widely used to purify liquid mixtures, including mixtures from industrial effluents [169]. Therefore, the contact time is not as long as in batch adsorption and hence equilibrium is not reached for a given adsorption. It can be said that the results from batch studies may not be accurate due to the reduced contact time for scale-up column adsorption [52]. Hence, it is crucial to study the practicality of using adsorbents in continuous mode. Batch adsorption studies provide important data and parameters for adsorbate removal, while passive samplers are an abiotic device used to monitor for chemicals or pollutants in an environmental medium [98,170,171]. 

Among adsorption techniques, batch adsorption is preferred by researchers in laboratory-scale studies because it consumes a small amount of material and is less time-consuming. In addition, studies of adsorption by equilibrium in batch mode allow adsorbent performance to be predicted prior to larger-scale application. The equilibrium study also provides important information about the effectiveness of a particular adsorbate-adsorbent system [172].

In this section, the attention is mainly on the application of chitosan-based nanocomposites as adsorbents for the removal of mercury from contaminated water by batch adsorption techniques and the removal of gaseous mercury from the atmosphere using the passive sampler adsorption method. 

### 6.1. Removal of Mercury from Wastewater Using Batch Adsorption Study

**Figure 8:**
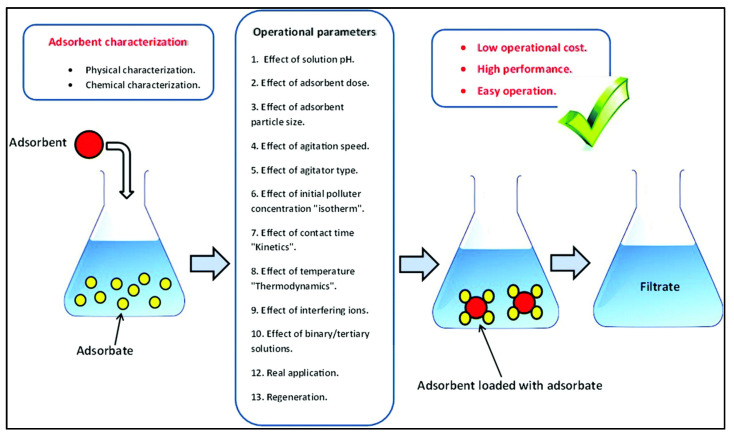
Schematic diagram of batch adsorption [174].

Batch adsorption experiments are common for laboratory scale studies, and are performed to evaluate the adsorption isotherms of metal ions (such as mercury) on the surface of the adsorbent (Figure 8) [105,173].

Thus, in this paper review, the focus is on chitosan-based polymer nanocomposites used as adsorbents in batch adsorption studies, as this is a promising method for water purification and environmental monitoring due to its simplicity, low cost, and potential to recycle the adsorbent nanomaterials [27,31,57,116,175,176,177]. The adsorption by functionalized chitosan-based polymer nanocomposites is a remarkable process due to the natural richness of the materials and non-toxic ability to exchange ions. The surface area has been shown to be the key factor influencing the adsorption capacity and efficiency of the adsorbent materials [107,110]. Moreover, chitosan also shows increased adsorption limits towards metal particles and other ionic atoms due to its different functional groups [178]. Investigations in one study showed that chitosan adsorbents have very high adsorption potentials for several types of heavy metals such as lead, chromium and mercury [179]. However, the adsorption of mercury has been demonstrated to be influenced by factors or parameters such as pH, adsorbent dosage, the contact time, adsorbate concentration, and temperature [180]. 

#### 6.1.1. pH

The effect of pH is critical in the adsorption process and is one of the most important process variables that can directly affect the uptake of Hg by adsorbents as it can affect the extent of Hg ionization as well as the surface properties of an adsorbent. For example, pH affects the overall charge on the adsorbent surface functional groups and their protonation, and it also affects the extent of ionization and speciation of the pollutants (Hg, Pb, and Cr) in the feed solution [181]. It has also been proved that the adsorption of pollutants such as metal ions is small at a pH value lower than the pH value of the zero-charge point due to the neutralization of the surface charge, while the pH value of the pollutant solution is higher than the pH value of the zero-charge point [111]. 

#### 6.1.2. Adsorbent Dosage

The adsorbent dose of solute adsorption increases with increasing concentration of an adsorbent because increasing adsorbent concentration results in increased active exchangeable adsorption sites. However, after increasing the adsorbent concentration, the total adsorption of solutes per unit weight of an adsorbent may decrease due to interference caused by the interaction of active sites of an adsorbent [182,183]. 

#### 6.1.3. Temperature

The temperature of the solution mainly affects the enlargement of adsorbents. At high operating temperature, it may be preferable for pollutant molecules to diffuse more quickly into the adsorbent, allowing for rapid bed saturation and short breakthrough time. Due to the increase in temperature, an endothermic process with an increase in adsorption capacity was observed [167]. 

#### 6.1.4. The Contact Time

The contact time can also influence the economics of the process and the adsorption kinetics. Therefore, the contact time is another performance-determining factor in the adsorption process and it significantly affects the adsorption process [184]. For instance, equilibrium for Pb^2+^ is reached after 60, 50 and 20 min for the initial concentration of 30, 20 and 10 ppm, respectively, when TiO_2_/MWCNTs or MWCNTs are used as adsorbents [111]. The study by Mamba and co-workers confirmed that equilibrium was reached after 60 min (for 4-CP) and 80 min (for Co^2+^) when pMWCNT-CD polymer was used at an initial concentration of 10 ppm [107].

#### 6.1.5. Initial Concentration of Pollutant

The influence of the initial concentration on the adsorption mechanism also depends on the type of adsorbent used. The study by Mamba et al. (2013) showed that the CD nanosponge polymer modified with functionalized CNTs performed much better than functionalized CNTs at low concentration (10 ppm). For example, removals by the polymer of 68% and 67% were recorded for lead, and cobalt, respectively [107,110]. It has also been reported that the concentration of anionic species in desorption solutions affects the regeneration of the adsorbent [185].

### 6.2. Removal of Mercury from the Atmosphere Using a Passive Sampler Adsorption Method

Passive air sampling (PAS) is another common strategy for gaseous Hg adsorption and measurement. PAS is widely used for environmental monitoring of mercury and other airborne pollutants [186]. Although passive air samplers (PAS) are not real-time monitoring systems, they overcome the limitations of other approaches such as active monitoring systems because they allow simultaneous spatial Hg measurement in different areas and thus create a map of the Hg concentration surrounding emission sources [187].

**Figure 9:**
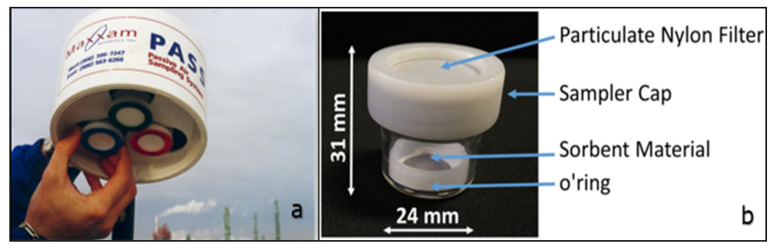
Field-used passive air samplers (PAS (**a**)) [189], and passive sampler with nylon filter and the glass jar with the polytetrafluoroethylene (PTFE) ring inside (**b**) [190], with permission from Elsevier, 2008.

Typically, in this technique, the adsorbent material in a PAS is located in a container protected by a membrane on a disc or columnar geometry surrounded by a cylindrical diffusion barrier as shown in Figure 9b [188]. The PAS consisted of a clear borosilicate vessel, a cap made of a nylon membrane for gas diffusion and particle stopping, a locking ring to keep the adsorbent membrane on the vessel bottom, and finally the adsorbent membrane on the quartz fibre substrate. The PAS used the axial diffusion path of the gaseous mercury through a diffusion membrane along a glass vessel (diffusion path) until it reached the adsorbing membrane (Figure 9a).

Samplers based on chitosan nanomaterial thin films or nanoparticles (NPs) [191,192] have been developed because of the strong electrostatic attraction mechanism. They are based on the second most abundant biopolymer and are inexpensive, re-usable and biodegradable; hence, they are used in passive mercury samplers for mercury uptake [61]. Moreover, chitosan-based polymer nanocomposites are used in water treatment because of their good flocculation properties, heavy metals adsorption (mercury) and ability to reduce the COD of organics; they have qualities such as fast removal rates and greater removal efficiency [193].

Furthermore, the uptake or performance of chitosan-based polymer nanocomposites is much more reliable when the nanostructured materials are incorporated into the matrix of the polymer. Incorporation of these nanostructured materials into the polymer matrix helps to control the shape, structural morphology, and size of the nanoparticles and improve the uptake efficiency of pollutants from the atmosphere [31,82,83,194,195,196,197,198,199].

## 7. Conclusions

According to various studies, chitosan has several natural benefits, including high porosity, biodegradability, structural integrity, and non-toxicity. The modification of chitosan with nanomaterials has been demonstrated to improve its properties and usefulness for various applications. In particular, functionalised chitosan-based composite materials (e.g., chitosan-based polymer nanocomposites) have attracted much attention as suitable adsorbents for dyes, oil spills, and heavy metal ions such as cobalt, mercury, and copper. Furthermore, these chitosan-based composite materials have been shown to possess excellent multifunctional properties resulting from the combination of the individual components. They have also been shown to improve adsorption efficiency due to the electron-rich functional groups present on the polymer backbone. In summary, these chitosan-based nanocomposites, produced by modifying functionalized chitosan with carbon nanostructured (carbon nanotubes) and nanoparticles (Ag, TiO_2_, ZnO, and S), have shown exceptional qualities, making them strong candidates as a promising method for environmental and wastewater remediation.

## 8. Future Perspectives

The research studies on the synthesis of chitosan-based polymer nanocomposites, mainly resulting from the modification of chitosan with CNTs, TiO_2_, Ag, ZnO or S nanoparticles, for application on environmental remediation to mercury have been scarce. Therefore, it is highly recommended that more investigations are undertaken in this area.

## Figures and Tables

**Table 1:** Mercury removal techniques with advantages and limitations.

Techniques	Advantages	Limitations	Reference
Adsorption	High efficiency Cost effective Availability of a wide selection of adsorbents High adsorption rates Easy to operate	Low selectivity	[16,17]
Solvent extraction	High Hg(II) selectivity	Time consuming Generation of secondary wastes Requires post-treatment step because of low separation efficiency	[17,18]
Chemical precipitation	Simple and convenient Not energy intensive	Large amounts of chemicals are needed Usually uses corrosive chemical Inefficient in wastewater with low concentrations of Hg ions Generation of sludge causing secondary contamination	[19,20,21]
Photocatalytic	Inexpensive depending on catalyst used	Formation of volatile Hg(0), which is also toxic and requires trapping	[22]
Flotation	Highly efficient High Hg selectivity Low detention periods	High initial capital costs	[23]
Ion exchange	Simple Cost-effective Efficient when thio based resins are used Tends to be cheap when natural zeolites are used	Requires a pretreatment step High cost of resins Resins used during the process require chemical regeneration that creates secondary pollution	[17,21]
Phytoremediation bio-remediation	Low cost Formation of less harmful by-products	For live microorganisms, the method is ineffective when metal concentration is high May affect plant growth and photosynthesis ability Sensitive to operational environment	[18]

**Table 2:** Properties comparison of different adsorbents for mercury remediation.

Materials	Advantages	Disadvantages	Reference
MnCe/zeolite	High thermal stability Superior and high activity	High cost operation	[84]
Ag-SBA-15	Multi-functional materials Outstanding regeneration capability Strong tolerance to complex flue gas High thermal and mechanical stability	High operating cost High operating temperatures.	[85]
Ag nanoparticles	High removal rate, Ultrahigh Ag atom utilization (150%) High selectivity and stability	High cost	[86]
MWCNTs	High efficiency	Low selectivity High cost	[87]
SiO_2_–TiO_2_	High stability	Poor photocatalytic activity	[88]
Activated carbon	Good adsorption capacity popular for the removal of pollutant from waste water.	They are costly (the higher the quality the greater the cost) Low selectivity	[29]

**Table 3:** Enhancement in modification and applications as a result of chitosan composite formation.

Chitosan-Based Nanomaterial	Techniques	Target Media (H_2_O or atm)	Removal Capacity	Reference
Sulfur-doped reduced graphene oxide@chitosan composite	Batch adsorption	H_2_O	0.125 to 6 μM Hg^2+^ with a detection limit of 1.6 nM.	[159]
Ch functionalized Au@S-g-C_3_N_4_	Passive sampling	atm	Limit of detection 0.275 nM	[160]
Chitosan/CNTs	Batch adsorption	H_2_O	148.7 mg/g (CS); 183.2 mg/g (MWCNT-COOH-impregnated CS beads); 167.5 mg/g (MWCNT-impregnated CS beads); and 172.7 mg/g (SWCNT-impregnated CS composite beads)	[161]
Thiol terminated chitosan capped silver nanoparticles (Mod-Ch-Ag NPs)	Batch adsorption	H_2_O	Detection limit is 5 ppb and response time is 5 s	[162]
Au-TiO_2_ nanoparticles/chitosan/gold (Au–TiO_2_ NPs/Ch/Au)	Passive sampler	atm	5.0–400.0 nM. In addition, the limit of detection is 1.0 nM with a 240 s preconcentration	[163]
Nano-SiO_2_-Crosslinked chitosan-nano-TiO_2_	Microwave-assisted sorption	H_2_O	8000 μmol g^–1^	[164]

Abbreviation: Ch—Chitosan; Au—Gold; TiO_2_—Titanium dioxide; CNTs—Carbon nanotubes; S—Sulfur; SiO_2_—Silicon dioxide; H_2_O—Water; atm—atmosphere.
